# Modular function of long noncoding RNA, COLDAIR, in the vernalization response

**DOI:** 10.1371/journal.pgen.1006939

**Published:** 2017-07-31

**Authors:** Dong-Hwan Kim, Yanpeng Xi, Sibum Sung

**Affiliations:** Department of Molecular Biosciences and Institute for Cellular and Molecular Biology, the University of Texas at Austin, TX, United States of America; Univ. Wisconsin, UNITED STATES

## Abstract

The long noncoding RNA COLDAIR is necessary for the repression of a floral repressor *FLOWERING LOCUS C* (*FLC*) during vernalization in *Arabidopsis thaliana*. The repression of *FLC* is mediated by increased enrichment of Polycomb Repressive Complex 2 (PRC2) and subsequent trimethylation of Histone H3 Lysine 27 (H3K27me3) at *FLC* chromatin. In this study we found that the association of COLDAIR with chromatin occurs only at the *FLC* locus and that the central region of the COLDAIR transcript is critical for this interaction. A modular motif in COLDAIR is responsible for the association with PRC2 *in vitro*, and the mutations within the motif that reduced the association of COLDAIR with PRC2 resulted in vernalization insensitivity. The vernalization insensitivity caused by mutant COLDAIR was rescued by the ectopic expression of the wild-type COLDAIR. Our study reveals the molecular framework in which COLDAIR lncRNA mediates the PRC2-mediated repression of *FLC* during vernalization.

## Author summary

Vernalization is an epigenetic response to winter cold necessary for the onset of flowering in spring. The epigenetically stable repression of a floral repressor, *FLC*, is a hallmark of the vernalization response in the model plant *Arabidopsis*. Vernalization-mediated repression of *FLC* is achieved by coordination among chromatin-modifying complexes and several long noncoding RNAs (lncRNAs). COLDAIR is an intronic lncRNA that is transcribed from the first intron of *FLC* and is necessary for the enrichment of PRC2 at the *FLC* locus. We found that COLDAIR is enriched only at *FLC* chromatin within the COLDAIR-enriched region. The mutations that disrupted the association of COLDAIR with PRC2 resulted in vernalization insensitivity. Therefore, our study demonstrates that epigenetic repression of FLC during vernalization involves the association of intronic long noncoding RNA COLDAIR with its target chromatin and a chromatin-modifying complex during vernalization.

## Introduction

Epigenetic regulation of gene expression in eukaryotes is coordinated by a series of molecular events, including chromatin modification, long noncoding RNAs (lncRNAs), RNA modification, and DNA modification [1–4]. The vernalization response in plants is one example in which epigenetic regulation of gene expression operates. In *Arabidopsis*, vernalization functions mainly through the stable repression of the gene encoding the floral repressor, FLOWERING LOCUS C (FLC), upon the exposure to an extended period of cold (i.e., winter cold). The repression of *FLC* by vernalization is accompanied by a series of changes in histone modifications at *FLC* chromatin. These changes include the removal of active histone markers, such as Histone H3 Lys 4 and Lys 36 trimethylation (H3K4me3 and H3K36me3, respectively), and histone acetylation [5–7], and the deposition of repressive histone markers, such as Histone H3 Lys 27 (H3K27me3) [5, 7–10]. In addition, several lncRNAs are transcribed from the *FLC* locus in response to vernalization, suggestive of functions in vernalization-mediated repression of *FLC* [11, 12].

Changes in chromatin modifications at *FLC* are mediated by a number of chromatin-remodeling complexes. In particular, genetic studies revealed that a core component of the evolutionarily conserved POLYCOMB REPRESSION COMPLEX 2 (PRC2), VERNALIZATION 2 (VRN2) is necessary for chromatin remodeling at *FLC* [13]. VRN2 is one of three Su(z)12 homologs in *Arabidopsis* [14]. Vernalization induces the expression of VERNALIZATION INSENSITIVE 3 (VIN3), which forms a complex with VRN2-containing PRC2 [8, 10, 15]. Two *Arabidopsis* homologs of E(z), CLF and SWINGER, also co-purify with VIN3 and VRN2 [10, 15]. VIN3 contains a plant homeo domain (PHD) finger motif [8], which binds to a range of modified histones [16]. Together with other VIN3-related proteins, PHD-PRC2 mediates the trimethylation of H3K27 at *FLC* chromatin [16]. The lncRNA COLDAIR also physically interacts with a component of PRC2, CLF, and is necessary for the increased enrichment of PRC2 at *FLC* chromatin during vernalization [11]. Similar interactions between PRC2 and other lncRNAs are necessary for Polycomb-mediated gene regulation in eukaryotes [17–21].

A growing body of evidence shows that lncRNAs are versatile regulators of epigenetic phenomena including dosage compensation, imprinting, and developmental gene expression in eukaryotes [22–24]. The molecular mechanisms by which lncRNAs exert their functions in various biological processes are not well understood. Among the best characterized lncRNAs are those that physically interact with PRC2 [18, 19, 21, 25]. In X-chromosome inactivation, both RepA and Xist noncoding RNAs bind to PRC2. RepA targets PRC2 to the Xist promoter and is associated with Xist upregulation; full-length Xist then binds and recruits PRC2 to the rest of the X chromosome [19, 26]. Xist also binds to another protein factor, YY1 (homolog of the fly Pho Polycomb protein), and forms an RNA-protein complex that is targeted in an allele-specific manner [27]. Similarly, HOTAIR lncRNA binds to multiple protein complexes, including PRC2 and LSD1, and functions as a scaffold for multiple protein complexes to coordinate chromatin modifications at both *cis*- and *trans*- targeted chromatin [18, 28]. Genome-wide identification of PRC2-binding noncoding RNAs suggests that a common RNA secondary structure may be necessary for the binding to PRC2 [17, 20, 29]. Other studies, however, have shown that mammalian PRC2 exhibits rather promiscuous binding to RNA, raising the question of whether specific structures of lncRNAs are critical for the PRC2-RNA interaction [30]. Nevertheless, there are clear examples of modular structures of lncRNAs with proteins [20, 31], although their biological significance remains to be addressed.

Here, we characterized the role of COLDAIR lncRNA in mediating *FLC* repression during vernalization. We mapped the regions of COLDAIR that interact with the *FLC* locus and with PRC2. COLDAIR was enriched only at *FLC* chromatin, and mutations that disrupted the association of COLDAIR with PRC2 resulted in vernalization insensitivity. Our data indicate that the conformation of COLDAIR is important in its function in the vernalization response.

## Results

### Interaction between COLDAIR and *FLC* chromatin

**Fig 1:**
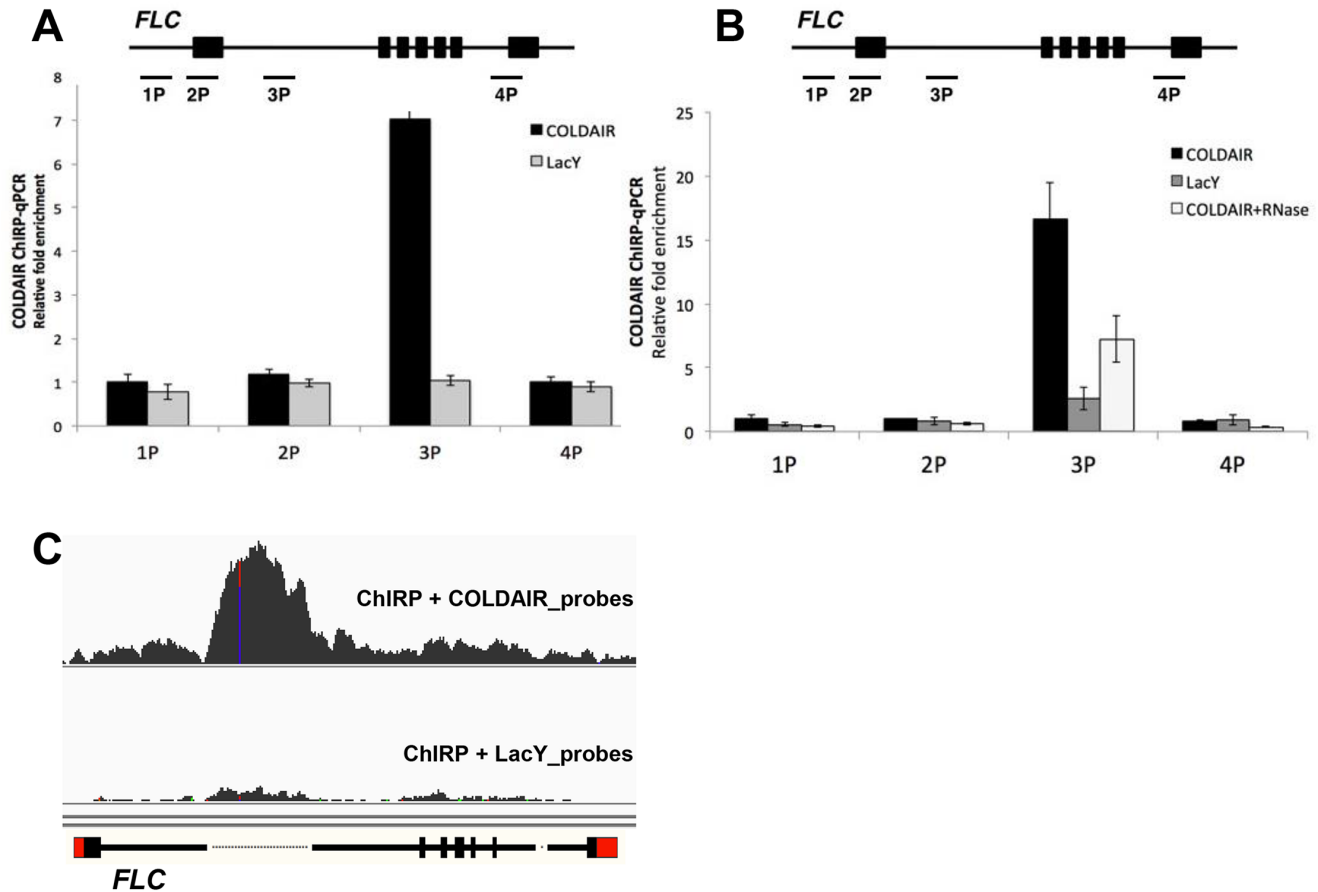
Detection of COLDAIR-associated chromatin by ChIRP. **(A)** (top) Schematic representation of *FLC* gene with regions of primer binding (P1, P2, P3, and P4) used in ChIRP detection indicated; the P3 binding site is within the COLDAIR coding area. (bottom) Plot of amount of DNA precipitated. Anti-COLDAIR probes retrieved a significant amount of DNA only in the P3 area (black), whereas anti-LacY probes showed no significant enrichment (grey). Means ± SD are shown (*n* = 3). **, *p*<0.01. **(B)** Detection of ChIRP result by qPCR after RNase treatment. Anti-COLDAIR probes showed significant enrichment in P3 area (black), which was reduced after RNase A and RNase H treatment of the chromatin prior to precipitation (white). Anti-LacY probes showed no enrichment (grey). Means ± SD are shown (*n* = 3). **, *p*<0.01, *, *p*<0.05. **(C)** Browser tracks of normalized ChIRP-Seq results with anti-COLDAIR probes (upper panel) and anti-LacY probes (lower panel).

Several methodologies have been used to locate lncRNA binding sites on chromatin in eukaryotes [32, 33]. We adopted the method known as Chromatin Isolation by RNA Purification (ChIRP) [33] to determine the genomic distribution of COLDAIR. Twenty-two biotinylated oligonucleotide probes complementary to the COLDAIR transcript were used to precipitate COLDAIR-associated chromatin (S1 Table). As a negative control, we also used probes complementary to the LacY transcript [33], which is similar to COLDAIR in length and nucleotide composition. We performed ChIRP from seedling samples that were vernalized for 20 days, the time at which the level of COLDAIR transcript peaks [11]. First, we analyzed ChIRP-precipitated DNA using quantitative PCR (ChIRP-qPCR) across *FLC* (Fig 1A). ChIRP-qPCR showed the peak enrichment at the COLDAIR transcribed region of *FLC* (Fig 1A). LacY probes did not show any biased enrichment at *FLC* regions, demonstrating the specificity of the ChIRP-qPCR assay. To exclude the possibility that the signal at the COLDAIR region was due to the hybridization between DNA and biotinylated COLDAIR probes, we treated ChIRP-precipitates with an RNase cocktail (RNase A/H) prior to the hybridization. RNase treatment resulted in the loss of peaked signal at COLDAIR region (Fig 1B), indicating that the observed enrichment is indeed mediated by hybridization between RNA and DNA probes.

The resolution of ChIRP-qPCR did not permit us to evaluate the relative distribution of COLDAIR-associated chromatin. To evaluate the relative level of enrichment of COLDAIR at a higher resolution we performed deep sequencing of ChIRP precipitates (ChIRP-Seq) as previously described [33]. ChIRP-Seq analysis showed strong signals within the COLDAIR region, with the sequencing reads peaking at around 480-530^th^ nucleotides from the COLDAIR transcription start site (TSS; Fig 1C and S1 Fig). Interestingly, several other regions within *FLC* showed relatively high enrichment compared to neighboring chromatin (S1 Fig). This rather broad association indicates the formation of higher-order of chromatin structure at *FLC* [34, 35]. Vernalization also triggers the enrichment of PRC2 and H3K27me3 at other members of *FLC* family genes [16]; however, we did not observe any significant enrichment for COLDAIR on *FLC*-related gene loci (S1 Fig) or elsewhere in the genome. Therefore, the association of COLDAIR appears to be specific to the *FLC* locus.

### Identification of the COLDAIR region that is necessary for the PRC2 association

**Fig 2:**
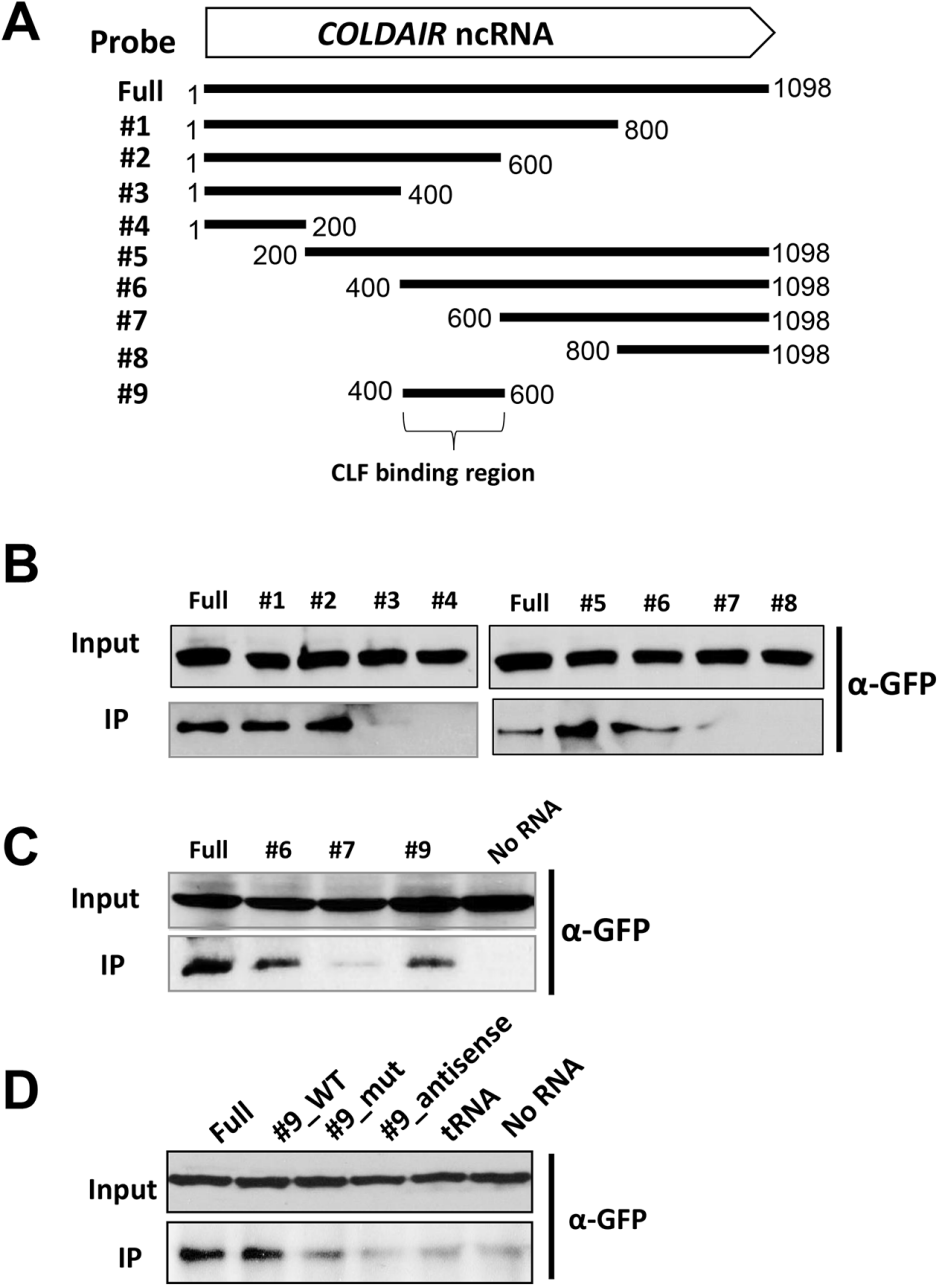
Mapping of CLF-interacting region of COLDAIR. **(A)** Schematic representation of full length and truncated transcripts of COLDAIR used in RNA binding assay. **(B~D)** RNA-binding assays using biotinylated RNAs and nuclear extracts of transgenic plants that express GFP-CLF. Anti-GFP antibody (ab290, Abcam) was used in western blot analyses. tRNA was used as a random RNA control (**D**).

Many lncRNAs interact with protein components through specific motifs that are formed by modular stem-and-loop structures in the RNA [17, 20, 29, 36, 37]. To identify the region of COLDAIR that mediates the interaction with PRC2, we utilized an *in vitro* RNA binding assay [11]. Nuclear extract from transgenic lines that express GFP-tagged CLF, a PRC2 component, were incubated with *in vitro* transcribed, biotinylated full-length and partial transcripts of COLDAIR (Fig 2A). Proteins associated with each RNA fragment were precipitated with streptavidin beads and GFP-tagged CLF protein was detected by western blot. From these experiments, we determined that only COLDAIR fragments containing nucleotides from 401 to 600 precipitate CLF-containing PRC2 (Fig 2B). In addition, the 200-base fragment of COLDAIR that only contains nucleotides 401 to 600 bases of COLDAIR was able to bind to CLF (Fig 2C and 2D).

### The role of the PRC2-binding motif of COLDAIR *in planta*

**Fig 3:**
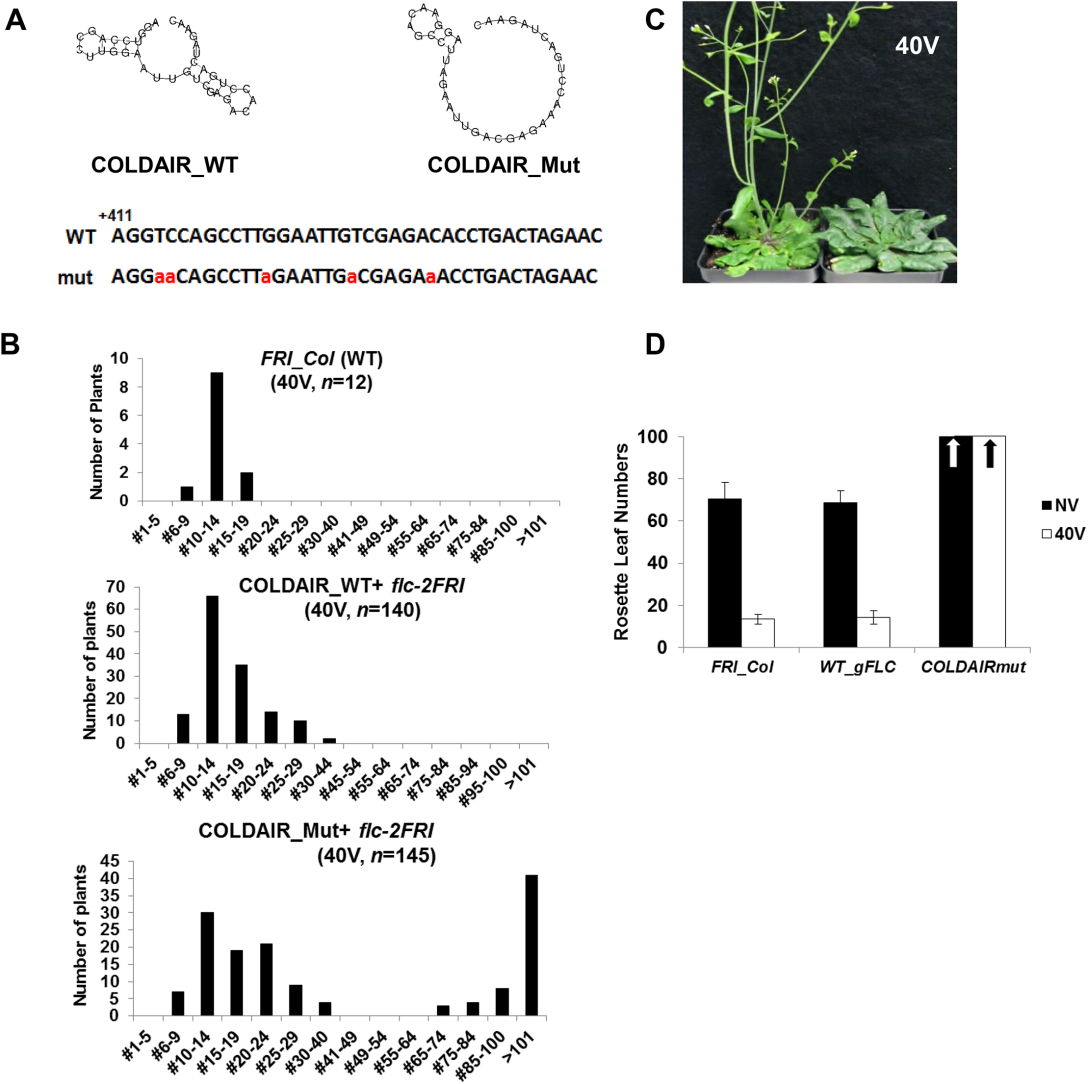
COLDAIR_Mut transgenic plants exhibit late flowering after vernalization. **(A)** Predicted secondary structures and sequences of wild-type COLDAIR and COLDAIR_Mut (http://rna.tbi.univie.ac.at/cgi-bin/RNAWebSuite/RNAfold.cgi). Mutated nucleotides are indicated in red. **(B)** Flowering time of wild-type and primary transgenic plants. Top) Flowering times of wild-type (*FRI_Col*) plants after 40 days vernalization. Middle) Flowering times of the primary transgenic lines carrying wild-type genomic *FLC* transgene (COLDAIR_WT) in *flc-2* after 40 days vernalization. Bottom) Flowering times of the primary transgenic lines carrying mutated genomic *FLC* transgene (COLDAIR_Mut) in *flc-2* after 40 days vernalization. *X*-axis: rosette leaf numbers at flowering. **(C)** Photographs of representative plants showing the flowering behaviors of *flc-2 FRI* transformed with the wild-type *FLC* transgene (left) and *flc-2 FRI* that express COLDAIR_Mut (right) after 40 days of vernalization. (**D)** Flowering time as number of rosette leaves on wild-type (*FRI_Col*), COLDAIR_WT, and COLDAIR_Mut plants that were non-vernalized (NV) and vernalized (40V). Arrows indicate the flowering time with more than 100 leaves.

Although our binding assay showed that a very specific region of COLDAIR is necessary for PRC2 binding, it was previously shown that PRC2 binds rather promiscuously to RNA molecules *in vitro* [30]. Within the 200-base fragment of COLDAIR that binds to CLF is a region predicted to form a distinctive stem-and-loop structure (Fig 3A and S2A Fig). Therefore, we evaluated the *in vivo* role of this predicted structure by introducing point mutations that should preclude formation of one of the predicted stem structures in the PRC2-binding region of COLDAIR (Fig 3A). To make these mutations, we utilized the *flc* deletion allele, *flc-2*. The *flc-2* allele contains a large deletion in *FLC* that includes COLDAIR [38]. We introduced five point mutations to disrupt the hairpin in the PRC2-binding motif of COLDAIR (referred as COLDAIR_Mut). As a control, we inserted the wild-type genomic construct into the *flc-2* mutants (referred as COLDAIR_WT; S2 Fig). Transgenic plants with the *FLC* transgene often vary in flowering time in part due to the dosage effect of *FLC* [38, 39], and thus we analyzed a large number of primary transgenic lines. Most transgenic lines with the wild-type COLDAIR (COLDAIR_WT) had a vernalization response comparable to that of non-transgenic wild-type plants (Fig 3B). On the other hand, about the half of transgenic lines with the COLDAIR mutant construct (COLDAIR_Mut) showed compromised vernalization response, flowering late even after vernalization (Fig 3B, 3C and 3D). This result suggests that a structure adopted by COLDAIR is important in regulation of the vernalization response. Transgenic plants showing early flowering compared to the wild-type plants even in the absence of vernalization exhibited lower levels of *FLC* expression, suggestive of inefficient transgene activity (S3 Fig). Several COLDAIR_Mut lines from late cohort transgenic lines were selected and used for further molecular analyses.

### The effect of the COLDAIR mutant on the vernalization-mediated *FLC* repression

**Fig 4:**
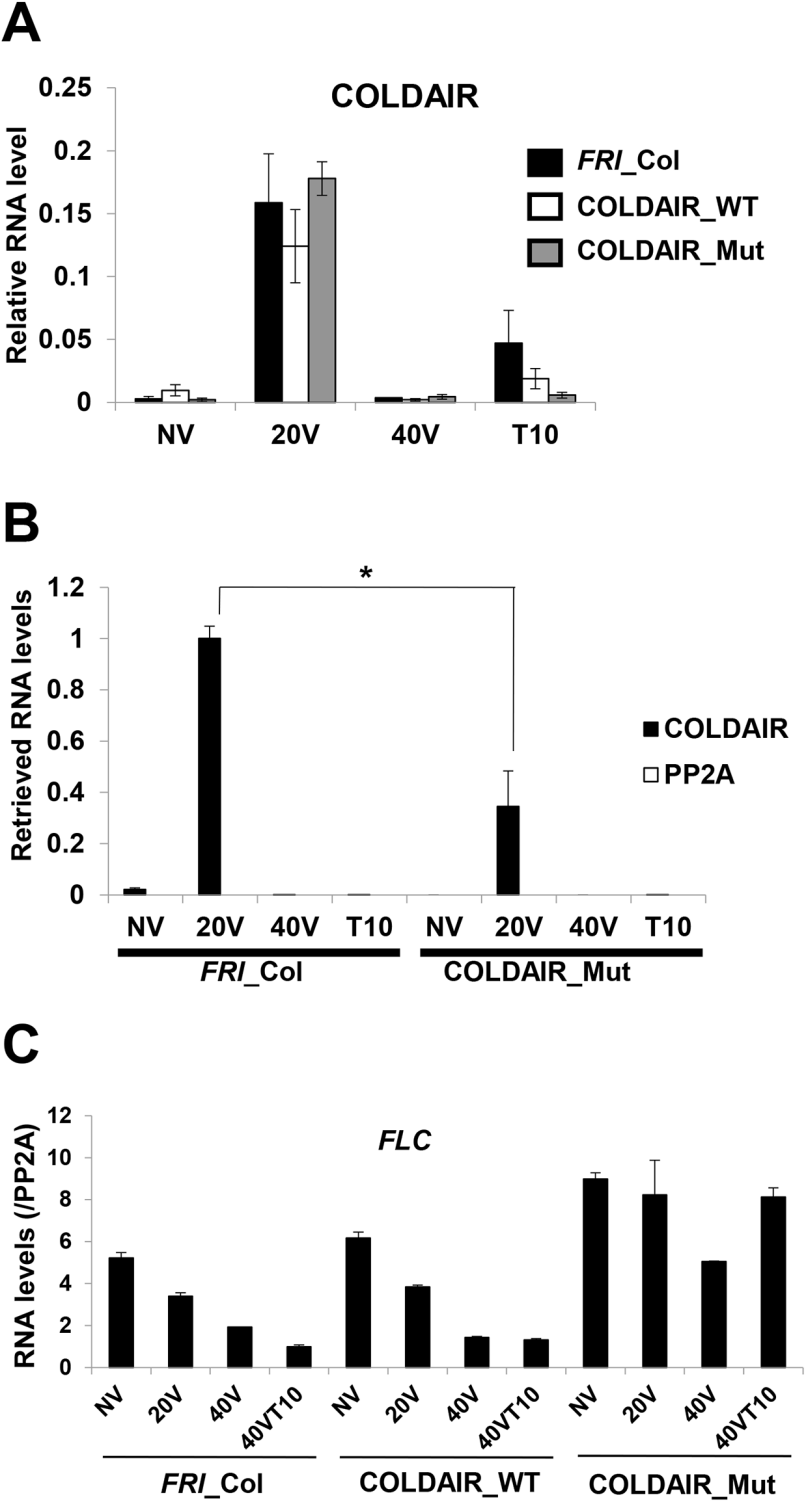
Stable repression of *FLC* by vernalization is impaired in transgenic lines that express COLDAIR_Mut. **(A)** Relative COLDAIR transcript amounts during the course of vernalization in wild-type (*FRI*_Col) plants, transgenic lines expressing COLDAIR_WT, and transgenic lines expressing COLDAIR_Mut. (**B)** Relative fold changes in COLDAIR RNA retrieved by RNA immunoprecipitation using anti-CLF antibody followed by qRT-PCR from wild type (*FRI*_Col) and COLDAIR_Mut plants. **(C)** Levels of *FLC* mRNA during the course of vernalization in the wild-type (*FRI*_Col) plants and transgenic lines expressing COLDAIR_WT and COLDAIR_Mut. Data plotted are means ± SD; *n* = 3; * *p*<0.1.

COLDAIR is transiently induced by vernalization [11], and thus we measured the level of COLDAIR expression in wild-type, COLDAIR_WT, and COLDAIR_Mut plants during the course of vernalization. Expression of COLDAIR was comparable among non-transgenic wild-type, COLDAIR_WT, and COLDAIR_Mut plants, and expression of COLDAIR peaked at 20-days of vernalization in all plants (Fig 4A). Therefore, the compromised vernalization response observed in COLDAIR_Mut is not due to the change in the level of COLDAIR expression.

It was demonstrated previously that the association of COLDAIR with PRC2 is most significant at 20 days of cold treatment [11]. We compared the relative levels of PRC2 association with COLDAIR by RNA immunoprecipitation (RIP) assays using the antibody against CLF. The amount of COLDAIR associated with PRC2 at 20 days of vernalization in COLDAIR_Mut was significantly reduced compared to the amount in wild-type (Fig 4B). This result indicates that the mutations that disrupt a modular structure of COLDAIR decrease the association of COLDAIR with PRC2 *in vivo*.

Reduced function of COLDAIR results in the unstable repression of *FLC* [11]. In both non-transgenic wild-type and COLDAIR_WT transgenic lines, the level of *FLC* mRNA expression was reduced during the cold treatment, and *FLC* mRNA expression remained repressed even after the vernalizing cold treatment (Fig 4C). In COLDAIR_Mut transgenic lines, the level of *FLC* was reduced during vernalizing cold treatment; however, the reduced level of *FLC* expression was not maintained once the plants were moved to warm temperature (Fig 4C). Instead, the level of *FLC* mRNA increased when plants were moved to normal growth temperature (22°C), indicating that the repression of *FLC* by vernalization is not maintained. This unstable repression of *FLC* is observed in other mutants in which the function of PRC2 is compromised [8, 9, 11, 40, 41]. These data are consistent with our hypothesis that a structure formed by COLDAIR is necessary for PRC2-mediated repression of *FLC*.

### The effect of the COLDAIR mutant on *FLC* chromatin

**Fig 5:**
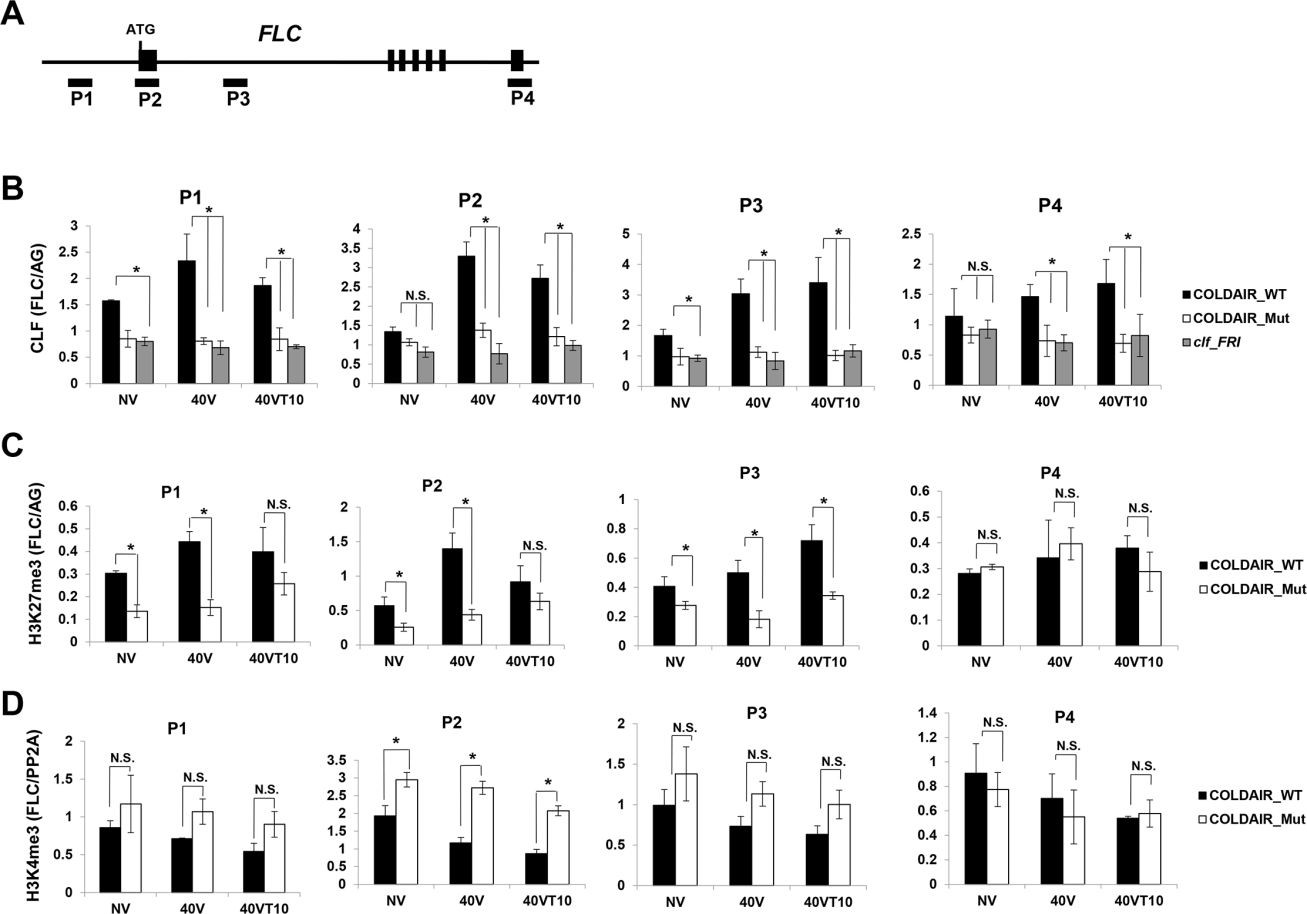
Vernalization-mediated histone modifications over *FLC* are impaired in COLDAIR_Mut line. **(A)** Schematic representation of *FLC* gene with regions of primer binding (P1, P2, P3, and P4) used indicated; the P3 binding site is within the COLDAIR coding area. **(B)** Occupancy of CLF at *FLC* chromatin in COLDAIR_WT and COLDAIR_Mut lines during the course of vernalization. **(C)** H3K27me3 at *FLC* chromatin in COLDAIR_WT and COLDAIR_Mut lines during the course of vernalization. **(D)** H3K4me3 at *FLC* chromatin in COLDAIR_WT and COLDAIR_Mut lines during the course of vernalization. Data plotted are means ± SD of qPCR; *n* = 4; * *p*<0.05.

COLDAIR is necessary for the enrichment of PRC2 at *FLC* by vernalization [11]. Our RIP analysis showed that the point mutations in COLDAIR_Mut lines resulted in the reduced association of PRC2 *in vivo* (Fig 4B). To address the effect of the reduced association of COLDAIR with PRC2, we examined the enrichment of PRC2 at *FLC* chromatin during the course of vernalization by chromatin immunoprecipitation (ChIP) followed by qPCR using the antibody against CLF (Fig 5A). The enrichment of CLF increased at the promoter and the first intron of *FLC* chromatin in COLDAIR_WT lines upon vernalization as expected (Fig 5B). However, increased enrichment of CLF by vernalization at *FLC* was substantially reduced in COLDAIR_Mut lines (Fig 5B). This result indicates that the association of COLDAIR with PRC2 is necessary for the increased enrichment of PRC2 at *FLC* chromatin during vernalization.

The level of H3K27me3, a histone modification mediated by PRC2, undergoes incremental enrichment at *FLC* chromatin upon vernalization [7, 10, 16]. The expected increase in enrichment of H3K27me3 at *FLC* chromatin was observed in COLDAIR_WT lines (Fig 5C). The overall level of H3K27me3 enrichment at *FLC* chromatin was lower in the COLDAIR_Mut than that in COLDAIR_WT seedlings (Fig 5C). Therefore, the reduced level of PRC2 enrichment at *FLC* chromatin was correlated with reduced enrichment of H3K27me3 across all vernalization time points in COLDAIR_Mut.

The enrichment of H3K4me3 at the TSSs of various loci correlates well with the level of transcription in many eukaryotes [42, 43]. H3K4me3 modification is considered an “active histone mark”. Consistent with this, we observed higher levels of H3K4me3 enrichment at *FLC* chromatin in COLDAIR_Mut lines, particularly at the TSS, than in COLDAIR_WT seedlings (Fig 5D). In addition, the level of H3K4me3 decreased during vernalization in COLDAIR_WT lines but not in COLDAIR_Mut lines (Fig 5D). Therefore, mutations in COLDAIR that reduce its association with PRC2 *in vivo* also compromise alterations in histone modifications at *FLC* that are triggered by vernalization.

### Restoring the vernalization response of COLDAIR_Mut lines by the expression of wild-type COLDAIR in *trans*

**Fig 6:**
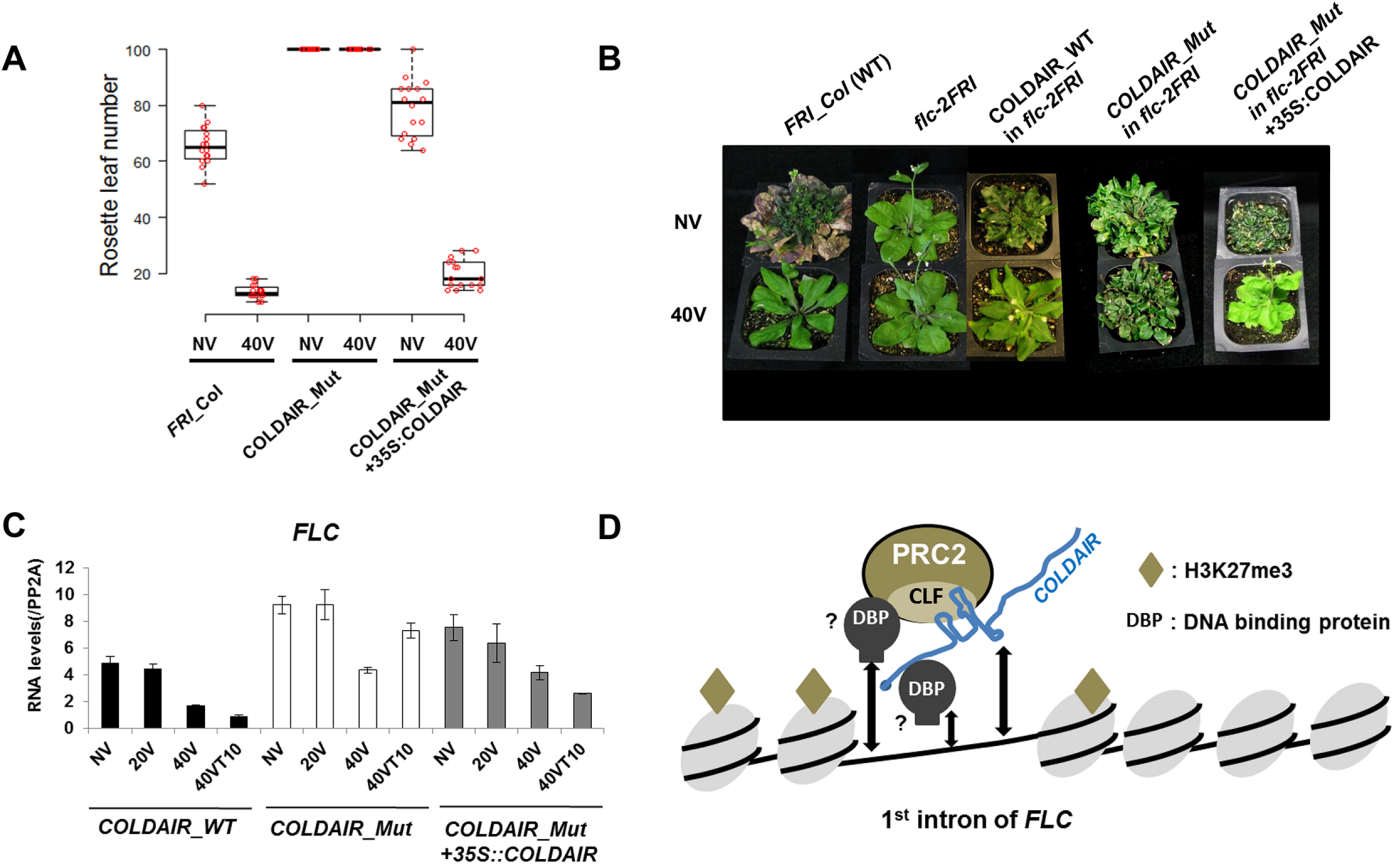
Ectopic expression of COLDAIR restores vernalization response in COLDAIR_Mut lines. **(A)** Flowering times of the wild-type plants (*FRI_Col*), COLDAIR_Mut plants, and and COLDAIR_Mut plants complemented with the 35S::COLDAIR at NV and 40V (*n* = 16). **(B)** Representative flowering behaviors of wild-type (*FRI_Col*) plants, *flc-2FRI*, COLDAIR_WT, COLDAIR_Mut, and COLDAIR_Mut complemented with 35S::COLDAIR without (NV) and with (40V) vernalization. **(C)** Levels of *FLC* mRNA during the course of vernalization in COLDAIR_WT, COLDAIR_Mut, and COLDAIR_Mut complemented with 35S::COLDAIR. **(D)** Schematic model of interactions of COLDAIR with the PRC2 complex over *FLC* during vernalization. A structured region of the COLDAIR transcript, which originates from the first intron of *FLC*, interacts with CLF-containing PRC2 complex during vernalization. Whether the interaction of COLDAIR-PRC2 complex with DNA is direct or other DNA-binding proteins (DBP) are involved remains to be determined.

Experiments described above do not exclude the possibility that mutations introduced to create the COLDAIR_Mut may have altered *cis* elements that do not necessarily function through the COLDAIR transcript. Therefore, we introduced a wild-type copy of COLDAIR into COLDAIR_Mut lines to test whether the wild-type COLDAIR could compensate for the reduced function of COLDAIR_Mut. The introduction of the wild-type COLDAIR driven by the 35S constitutive promoter into COLDAIR_Mut lines resulted in accelerated flowering after vernalization (Fig 6A and 6B). This provided additional evidence that a lack of structure in the COLDAIR transcript itself is responsible for the reduced vernalization response in COLDAIR_Mut lines. Accelerated flowering is not due to a non-specific repressive effect on *FLC* transcription by ectopically expressed wild-type COLDAIR, as 35S-driven COLDAIR expression did not alter the flowering time in COLDAIR_Mut without vernalization (Fig 6A and 6B). The repression of *FLC* by vernalization is restored, however, by 35S-driven COLDAIR expression (Fig 6C). Unlike COLDAIR_Mut lines, in which the repression of *FLC* was not stably maintained, the repression of *FLC* was stably maintained after vernalization treatment in COLDAIR_Mut lines in which wild-type COLDAIR is expressed, consistent with the functional complementation of COLDAIR_Mut by the ectopic expression of wild-type COLDAIR. These results indicate that COLDAIR triggers the repression of *FLC* in *trans*. A similar *trans* repression by Xist lncRNA is observed in the PRC2-mediated silencing of the mammalian X chromosome [26, 27].

It should be noted that the ectopic expression of COLDAIR does not result in early flowering comparable to vernalized plants. This result was expected as other cold-induced events (i.e., the induction of VIN3) must occur to achieve the full extent of *FLC* repression by vernalization. We also introduced the mutant COLDAIR into COLDAIR_Mut lines (S4 Fig) and observed that the restoration of vernalization response was less effective compared to that induced by the wild-type transgene, consistent with the less effective binding of the mutant COLDAIR to CLF *in vivo* (Fig 4B).

## Discussion

In this report, we characterized the role of the lncRNA COLDAIR in interactions with PRC2 and the *FLC* locus. There are other functionally similar lncRNAs known to be associated with chromatin and to function in gene regulation [44]. lncRNAs can act at the site where they are transcribed (*cis* acting). Some of these lncRNAs are involved in co-transcriptional regulation as implicated for COOLAIR lncRNA in vernalization [6]. lncRNAs may also serve as *trans*-acting factors to regulate genes outside their transcription unit. Many models of lncRNA function fall between these two extremes [45, 46]. ChIRP-Seq analysis showed that COLDAIR transcripts associate with *FLC* chromatin. The interactions of COLDAIR transcripts with *FLC* chromatin do not occur throughout the region from which COLDAIR is transcribed. Instead, there is a clear peak of concentrated enrichment of COLDAIR at *FLC* chromatin (Fig 1A, 1B and 1C). This indicates that COLDAIR forms a local contact with the *FLC*. The ChIRP-Seq data do not necessarily suggest that COLDAIR directly binds to *FLC* DNA because crosslinked chromatin was precipitated using oligonucleotide probes hybridized to COLDAIR.

Various mechanisms for interactions between lncRNAs and the chromatin-modifying machinery have been proposed [36, 47–49], including the idea that lncRNAs can act through base paring or triplex formation with DNA or RNA [50–52]. Alternatively, as in the case for the roX lncRNA, the specificity could be directed by proteins [53]. However, our ChIRP-Seq data do not exclude the possibility of the involvement of other protein components in the recruiting PRC2 to the *FLC* locus. There are other examples in which both proteins and lncRNAs coordinate to target chromatin-modifying complexes into the target chromatin [27, 52]. Only the small fraction of COLDAIR is used for the interactions with PRC2 and *FLC* chromatin. There are lncRNAs that serve as scaffolds for multiple protein components [27, 28, 36]. Whether the other parts of COLDAIR can serve as functional motifs (i.e., through the interaction with other protein complexes) remains to be determined.

COLDAIR is associated with only *FLC* chromatin, therefore it is reasonable to speculate that COLDAIR acts in *cis*; however, wild-type COLDAIR can rescue the COLDAIR mutant phenotype when expressed in *trans*. Therefore, our data indicate that co-transcriptional regulation is not necessary for COLDAIR function. Likewise, there are other examples of *trans* repression by lncRNAs, including Xist in mammals [27]. This predicts that the RNA-protein complexes can be formed before they are enriched at their target loci.

lncRNA structure has been implicated to be an integral part of the function of lncRNAs, such as HOTAIR and Xist lncRNAs [20, 31, 54–57]. However, the biological importance of these structures is less understood. In case of PRC2-binding lncRNAs, there have been reports that show the promiscuous binding of RNA by PRC2 [30, 58], making it difficult to evaluate the structural importance of lncRNAs. Here we showed that a stem-loop in COLDAIR is important for its interaction with the PRC2 complex. Severe defects in vernalization response of COLDAIR_Mut lines demonstrate the biological importance of the predicted RNA structures. Our current model is that COLDAIR base pairs with the *FLC* DNA and that a structured region of COLDAIR interacts with a component of the PRC2 complex to form RNA-protein complex at the *FLC* locus (Fig 6D). Our work provides insight into the mechanism by which COLDAIR interacts with a chromatin-modifying complex and its target chromatin.

## Materials and methods

### Chromatin isolation by RNA purification (ChIRP)

The ChIRP protocol was adapted from Chu et.al. [33]. Seedlings were crosslinked in 1% formaldehyde solution by vacuum infiltration in 4 ^o^C for 25 minutes. Crosslinking was terminated by adding 0.125 M glycine with vacuum infiltration for another 5 minutes. Crosslinked seedlings were ground to a fine power. About 1 ml of ground powder was suspended in 10 ml buffer 1 (0.4 M sucrose, 10 mM Tris-HCl, pH 8.0, 10 mM MgCl_2_, 5 mM beta-mercaptoethanol), and samples were put on ice for 10 min. The samples were then filtered through two layers of Miracloth, centrifuged at 2,000 *g* at 4°C for 20 minutes. Pellets were resuspended in 1 ml buffer 2 (0.25 M sucrose, 10 mM Tris-HCl, pH 8.0, 10 mM MgCl_2_, 1% Triton X-100, 5 mM beta-mercaptoethanol), centrifuged at 15,000 *g* at 4°C for 10 minutes. Pellets were suspended in 300 μl buffer 3 (1.7 M sucrose, 10 mM Tris-HCl, pH 8.0, 2 mM MgCl_2_, 0.15% Triton X-100, 5 mM beta-mercaptoethanol), layered on top of another 300 μl fresh buffer 3, centrifuged at 15,000 g at 4°C for 1 hour. These pellets were resuspended in 300 μl lysis buffer (50 mM Tris-HCl, pH 8.0, 10 mM EDTA, 1% SDS), sonicated (15 s ON/60 s OFF) until DNA was fragmented into 200–500 bp pieces. After centrifugation at 15,000 *g*, the chromatin in the supernatant was diluted with 2 volumes of hybridization buffer (500 mM NaCl, 1% SDS, 100 mM Tris, pH 7.0, 10 mM EDTA, 15% formamide). Probes (100 pmol), designed at http://www.singlemoleculefish.com, were added to 3 μl of diluted chromatin, and incubated by end-to-end rotation in 4°C overnight. For the RNAse A/H treatment to ascertain the reduction of the signal due to DNA-probe hybridization (Fig 2B), treatment with a cocktail of 100 μg/ml RNase A and 0.1 U/μl RNase H at 37°C with end-to-end rotation for 2 hour was performed prior to the addition of the probes. Streptavidin-magnetic C1 beads (Roche) were washed three times in lysis buffer, blocked with 500 ng/μl yeast total RNA and 1 mg/ml BSA for 1 hour at room temperature, washed again in lysis buffer before resuspended in original volume. To each sample was added 50 μl washed beads, and samples were mixed at 4°C for another 2 hours before capture by magnets. Captured beads were washed five times with wash buffer (2xSSC, 0.5% SDS), resuspended in 3x original volume DNA elution buffer (50 mM NaHCO_3_, 1% SDS, 200 mM NaCl) with a cocktail of 100 μg/ml RNase A and 0.1 U/μl RNase H at 37°C with end-to-end rotation. Chromatin was reverse-crosslinked at 65°C overnight. DNA was extracted with phenol:chloroform:isoamyl alcohol and subjected to downstream qPCR or high-throughput sequencing.

### Sequencing analysis

Sequencing libraries were constructed using the NEBNext ChIP-Seq library prep kit and sequenced on either an Illumina HiSeq 4000 or an Illumina Nextseq 500 with read length at 50 bp (ChIRP). Raw reads were mapped to reference genome (TAIR10) with Bowtie2. Mapped reads were normalized using Deeptools and visualized using IGV. Genome-wide read density profiles were constructed using the Deeptools plotProfile function. TSS and TES regions were extracted from the TAIR10 reference genome with mitochondria and plastid genes removed.

### Plant materials and growth conditions

All plants used in this study were *Columbia FRI*^*sf2*^ background unless otherwise specified. Seeds were germinated and seedlings were grown under short day (SD) conditions (8 h light and 16 h dark), then subjected to cold (4°C) for vernalization as previously described [59]. Samples were harvested for experiments immediately after the cold treatment (40V) or were grown for 10 days more at 22°C under SD conditions. Flowering time was measured by counting rosette leaf numbers at a bolting stage under the long day (16 h light, 8 h dark) condition.

### Transgenic plants

Genomic sequence spanning the promoter (1.7 kb), coding (6 kb) and 3’ (0.7 kb) region of *FLC* was amplified by PCR. This *FLC* fragment was cloned into the pPZP211 binary vector and used as a template to produce mutated forms of the genomic *FLC* fragment using site-directed mutagenesis with pairs of mutagenic primers and flanking primers. Mutated fragments were cloned into pPZP211 vector. Both WT and mutated forms of genomic *FLC* constructs were transformed into *Agrobacterium tumefaciens* GV3101 strain and transformed into *flc-2 FRI-Col* plants. Full-length COLDAIR was cloned into pENTR vector (Invitrogen) and then transferred into pEARLEY100 vector using LR clonase (Invitrogen). Sequences were confirmed and used for complementation in COLDAIR_Mut transgenic lines.

### RNA expression analysis

Total RNA was extracted from whole seedling plants using TRIzol (Invitrogen). Samples were treated with DNase (Promega) for 30 minutes at 37°C to eliminate contaminating genomic DNA. RNA (5 μg) was used for synthesis of first strand cDNA using random primers or oligo-dT and then used for real-time qRT-PCR analyses with Maxima SYBR green master mix (Thermo Scientific) on a ViiA 7 real-time system (Life Technologies). Relative transcript levels were normalized by comparing to levels of *PP2A* as previously described [16]. For COLDAIR, total RNA were used for first strand cDNA syntheses using Gene Link random primers with the Promega M-MLV System. Anchor primers AAP and AUAP (Invitrogen) and gene-specific reverse primers (COLDAIR_1R and COLDAIR_2R) were used for detection. Primer sequences are listed in S2 Table.

### RNA binding assay

Full-length and fragments of COLDAIR were amplified by PCR and then cloned into pENTR-D-TOPO (Invitrogen). The mutated fragment spanning nucleotides 401 to 600 of COLDAIR was amplified by PCR using site-directed mutagenesis method. Each insert sequence was confirmed by sequencing. Primer sequences used to produce each fragment are listed in S2 Table. Plasmids containing full-length and truncated fragments were digested with *Not*I to linearize the plasmid. Biotinylated RNA was prepared by *in vitro* transcription using the Biotin RNA Labeling Mix (Roche) and T7 RNA polymerase (Roche). Samples were treated with RNase-free DNase (Promega) and purified using RNeasy Mini Kit (Qiagen). Biotinylated RNA (3 μg) was incubated with nuclear extract from GFP-CLF transgenic plants in pull-down buffer (50 mM Tris-HCl, pH 7.5, 100 mM NaCl, 2 mM DTT, 0.05% NP-40, 40 U/ml RNase-OUT RNAse inhibitor (Invitrogen), and a Roche protease inhibitor tablet) at 4°C. Streptavidin agarose beads (Roche) were added to each binding reaction, and samples were rotated overnight at 4°C. Bead were washed at 4°C five times using pull-down buffer and were boiled in SDS loading buffer, and then loaded onto SDS-PAGE gel. Blots were analyzed using anti-GFP antibody (ab290, Abcam).

### Chromatin immunoprecipitation (ChIP) assay

Seedling samples were crosslinked in 1% formaldehyde-containing buffer and then ground in liquid nitrogen and used for ChIP experiments as previously described [59]. ChIP data were quantified by qPCR, then the IP/Input ratios were calculated for each primer set. The data across multiple primer sets and time-points were normalized by comparing to the IP/INPUT ratios of internal reference genes *AG* (At4g18960) for H3K27me3 and *CLF* or *PP2A* (At1g13320) for H3K4me3 ChIP experiments. The value of reference loci in WT samples at NV condition was set to 1. Data are represented by the average and SEM of two technical replicates of two biological replicates (*n* = 4). qPCR reactions were performed using ViiA^TM^ 7 Real-Time PCR System (Life Technologies). Antibodies used in this study were anti-H3K27me3 (ab6002, Abcam), anti-H3K4me3 (ab1012, Abcam), and anti-CLF [35, 60]. Primer sequences are given in S2 Table.

### RNA immunoprecipitation (RIP) assay

Whole seedlings were harvested and crosslinked. Crosslinked samples were ground in liquid nitrogen, then resuspended in nuclei isolation buffer (0.25 M sucrose, 5 mM PIPES, pH 8.0, 5 mM MgCl_2_, 85 mM KCl, 15 mM NaCl, 1% Triton X-100, 40 U/ml RNaseOUT (Invitrogen), 1 mM PMSF, 1 tablet of Roche protease inhibitor cocktail) and placed on ice for 15 minutes. The crude nuclear fraction was centrifuged at 15,000 *g* for 10 minutes at 4°C. Each pellet was resuspended in lysis buffer (50 mM HEPES, pH 7.5, 150 mM NaCl, 1% Triton X-100, 1 mM EDTA, 0.1% sodium deoxycholate, 1% SDS, 1 mM PMSF, 40 U/ml RNaseOUT (Invitrogen), 1 tablet of Roche protease inhibitor cocktail) and was sonicated five times, each 15 seconds. Sonicated lysates were centrifuged at 15,000 *g* for 15 minutes at 4°C. Supernatants were transferred to new tubes and 50 μl protein A agarose beads were added (Roche). Samples were rotated for 30 minutes at 4°C. Potein A agarose beads were pelleted. Each supernatant was diluted to 1 ml with dilution buffer (1.1% Triton X-100, 1.2 mM EDTA, 16.7 mM Tris-HCl, pH 8.0, 167 mM NaCl, 40 U/ml RNaseOUT (Invitrogen), 1 tablet of Roche protease inhibitor cocktail). Of each sample, 10% was reserved as the “input” RNA sample and frozen at -80°C. Anti-CLF antibody was added to each sample, and tubes were rotated overnight at 4°C. Samples were sequentially washed using low-salt, high-salt, LiCl, and TE buffers (pH 8.0). To each sample was added 100 μl elution buffer (1% SDS, 0.1 M NaHCO_3_, 40 U/ml RNaseOUT (Invitrogen)), and samples were incubated for 10 minutes at room temperature. The elution step was repeated once. Eluted samples were treated with 20 μg proteinase K and incubated for 1 hour at 42°C. To reverse crosslinking, each sample was treated with 10 μl 5 M NaCl and incubated for 1 hour at 65°C. RNA was purified using TRIzol (Invitrogen) according to manufacturer’s instruction. After treatment with RQ1 DNase (Promega) for 25 minutes at 37°C, RNA was used for first strand cDNA syntheses using random primers (Gene Link) with the M-MLV System (Promega). COLDAIR RNA levels were normalized to levels of *PP2A* mRNA. qRT-PCR reactions were performed in at least two biological replicates and two to three technical replicates on a ViiA 7 real-time system (Life Technologies). Primer sets used for qRT-PCR analyses are listed in S2 Table.

## Supporting information

S1 Table Sequences of probes used in ChIRP analysis. (PDF)

S2 Table Sequences of primers used in this study. (PDF)

S1 Fig Extended browser tracks of ChIRP-Seq. ChIRP-Seq read distribution around *FLC* and *MAF* family members (*MAF1*-*MAF5*).
(JPG)

S2 Fig Predicted structure of COLDAIR. **(A)** The sequence of COLDAIR. Sequences that are predicted to form a stem-and-loop structure within 401–600 nucleotide region of COLDAIR are indicated by bold characters. **(B)** Predicted structures of COLDAIR_WT and COLDAIR_Mut. Boxed area in COLDAIR_WT shows the region shown in Fig 3A. The structural models were predicted using the *RNA_fold* algorithm (http://rna.tbi.univie.ac.at/cgi-bin/RNAWebSuite/RNAfold.cgi). (**C)** Schematic of constructs used to create COLDAIR_WT (left) and COLDAIR_Mut plants (right). These constructs are introduced into *flc-2FRI*_Col mutants.
(JPG)

S3 Fig Analysis of transgenic lines. The level of *FLC* expression of 10 randomly selected T2-pools of transgenic lines carrying the mutant COLDAIR (COLDAIR_Mut) in *flc-2* mutant background and the wild-type COLDAIR (COLDAIR_WT) at the second generation (T2) compared to the non-transgenic (*flc-2FRI*). Due to the *FLC* transgene variability, transgenic lines were grouped together based on their flowering time before vernalization.
(JPG)

S4 Fig Analysis of transgenic lines of mutated COLDAIR. Number of COLDAIR_Mut plants complemented with the wild-type and mutant 35S::COLDAIR that flowered in NV (**A**) and 40V (**B**) conditions. *X*-axis: rosette leaf number at flowering. (**C**) flowering time of Primary transgenic plants of COLDAIR_Mut+35S-COLDAIR_WT (*n = 9*) and COLDAIR_Mut+35S-COLDAIR_Mut (*n = 9*) in 40V condition. Plants showing >80 leaves were excluded from statistical analysis because it is considered to be non-flowering. Data plotted are means ± SD; * *p*<0.5.
(JPG)
