# Supplementary material for: Modular function of long noncoding RNA, COLDAIR, in the vernalization response
Source: PLoS Genet. 2017 Jul 31;13(7):e1006939. doi: 10.1371/journal.pgen.1006939 (PMC5552341; doi:10.1371/journal.pgen.1006939)
Supplement: S1 Table — (PDF) [file pgen.1006939.s001.pdf]

Supplementary Table S1. Sequences of probes used in ChIRP analysis.

Name	5'-3' sequence	Target
CA1	caattttaacgaataggaac	COLDAIR tilling
CA2	tgacctagaagatccaacaa	COLDAIR tilling
CA3	tcacaacaaaagattgaggt	COLDAIR tilling
CA4	gcgactaagacagataaaaa	COLDAIR tilling
CA5	gaatgacaatttccatgaca	COLDAIR tilling
CA6	aatatatcttccatagaagg	COLDAIR tilling
CA7	tgggattttcaatttcctag	COLDAIR tilling
CA8	agtaggaaacttatcacaca	COLDAIR tilling
CA9	gatccgtaccaaagaggttg	COLDAIR tilling
CA10	agttttctgcccttgaagtt	COLDAIR tilling
CA11	gggttgtagtagacactaca	COLDAIR tilling
CA12	actgtgaggttccaatttcc	COLDAIR tilling
CA13	atgcatcaagtgagaatcgg	COLDAIR tilling
CA14	ccggtcttccattttgttat	COLDAIR tilling
CA15	tctagtcaggtgtctcgaca	COLDAIR tilling
CA16	atggaccgagtcttagacaa	COLDAIR tilling
CA17	cgttctaaaaggcttcttct	COLDAIR tilling
CA18	gcggtaaccagataaccata	COLDAIR tilling
CA19	aacctaagtatgcaaaaaga	COLDAIR tilling
CA20	ttttttccagcgatagagct	COLDAIR tilling
CA21	tgttaatagatatcagtcct	COLDAIR tilling
CA22	acgagaaaacttttcggatt	COLDAIR tilling
LY1	ccgaacatccaaaagtttgt	control LacY tilling
LY2	gaaaaacgggaagtaggctc	control LacY tilling
LY3	acagcggttggaataatagc	control LacY tilling
LY4	cacagcaggtatttgcgcag	control LacY tilling
LY5	aaataaagaacggcgcaaac	control LacY tilling
LY6	cgatcctactaaaatgttgt	control LacY tilling
LY7	ggcgttaaaacaaaagccta	control LacY tilling
LY8	cgcgcgaccaaattcgaaat	control LacY tilling
LY9	tgaacatgatgccgacaatc	control LacY tilling
LY10	catccgttttggcgaaaaag	control LacY tilling
LY11	ctaaatgccgaatggttggc	control LacY tilling
LY12	gccaataacatacagtgaca	control LacY tilling
LY13	aaattagcaaactgttggtc	control LacY tilling
LY14	gtaattcgcccattgtcgtt	control LacY tilling
LY15	cagcagggcgtttttcccac	control LacY tilling

LY16	aacgatgagccaataatacg	control LacY tilling
LY17	cagcaggaacggtacttcaa	control LacY tilling
LY18	aaaaacgcacttcaaactgg	control LacY tilling
LY19	atcgccagttgcttaaagaa	control LacY tilling
LY20	cagacccagcaccagataag	control LacY tilling
LY21	gggccgctaagcgtgaacac	control LacY tilling
LY22	gacttcattcacctgacgac	control LacY tilling
