# Supplementary material for: Modular function of long noncoding RNA, COLDAIR, in the vernalization response
Source: PLoS Genet. 2017 Jul 31;13(7):e1006939. doi: 10.1371/journal.pgen.1006939 (PMC5552341; doi:10.1371/journal.pgen.1006939)
Supplement: S2 Table — (PDF) [file pgen.1006939.s002.pdf]

Supplementary Table S2. Sequences of primers used in this study.

Name	Sequence	Usage
FLC_qF	gccaagaagaccgaactcatgttga	qRT-PCR
FLC_qR	caaccgccgatttaaggtggcta	qRT-PCR
PP2A_qF	tatcggatgacgattcttcgtgcag	qRT-PCR
PP2A_qR	gcttggtcgactatcggaatgagag	qRT-PCR
COLDAIR_1R	cacgttctaaaaggcttcttctttattaaatc	RACE_qPCR
COLDAIR_2R	caaatcctaacaaagtatgcatcaagtgag	RACE_qPCR
COLDAIR_F	ggccacgcgtcgactagtac	RACE_qPCR
COLDAIR_R	agtagacactacaccagattcaattttgac	RACE_qPCR
FLC_P1_F	cgtgagtccgcccctgatagc	ChIP
FLC_1P_R	ggaccaaaccaaacctacaaagactttc	ChIP
FLC_P2_F	cttagtatctccggcgacttgaacc	ChIP
FLC_2P_R	gcgtcacagagaacagaaagctga	ChIP
FLC_P3_F	acacaacctttgtatcttgtgtcttttg	ChIP
FLC_P3_R	agtagacactacaccagattcaattttgac	ChIP
FLC_P4_F	gtgaatagtgattttgacctatgattatcgtacag	ChIP
FLC_P4_R	ggtggctaattaagtagtgggagagtcac	ChIP
AG_F	gtgaaacaaattttcctgcagaatgtcact	ChIP & RIP
AG_R	agtttttgaggcactaaaatctttgggtaaatc	ChIP& RIP
PP2A_F	agcctttatacccgattgctgtgcttatcg	ChIP & RIP
PP2A_R	cctctcctctccaagagcacgagc	ChIP& RIP
COLDAIR_RIP_F	tacaaccctccaatataataaccaaatggttg	RIP_qRT_PCR
COLDAIR_RIP_R	cacgttctaaaaggcttcttctttattaaatc	RIP_qRT_PCR
COLDAIR_OE_F	cacctttgttcctattcgttaaaattgacaatccacaacctc	35S:COLDAIR
COLDAIR_OE_R	aacatatacgagaaaacttttcggatttttcaatgaacc	35S:COLDAIR
