# Supplementary figures and images for: Modular function of long noncoding RNA, COLDAIR, in the vernalization response
Source: PLoS Genet. 2017 Jul 31;13(7):e1006939. doi: 10.1371/journal.pgen.1006939 (PMC5552341; doi:10.1371/journal.pgen.1006939)

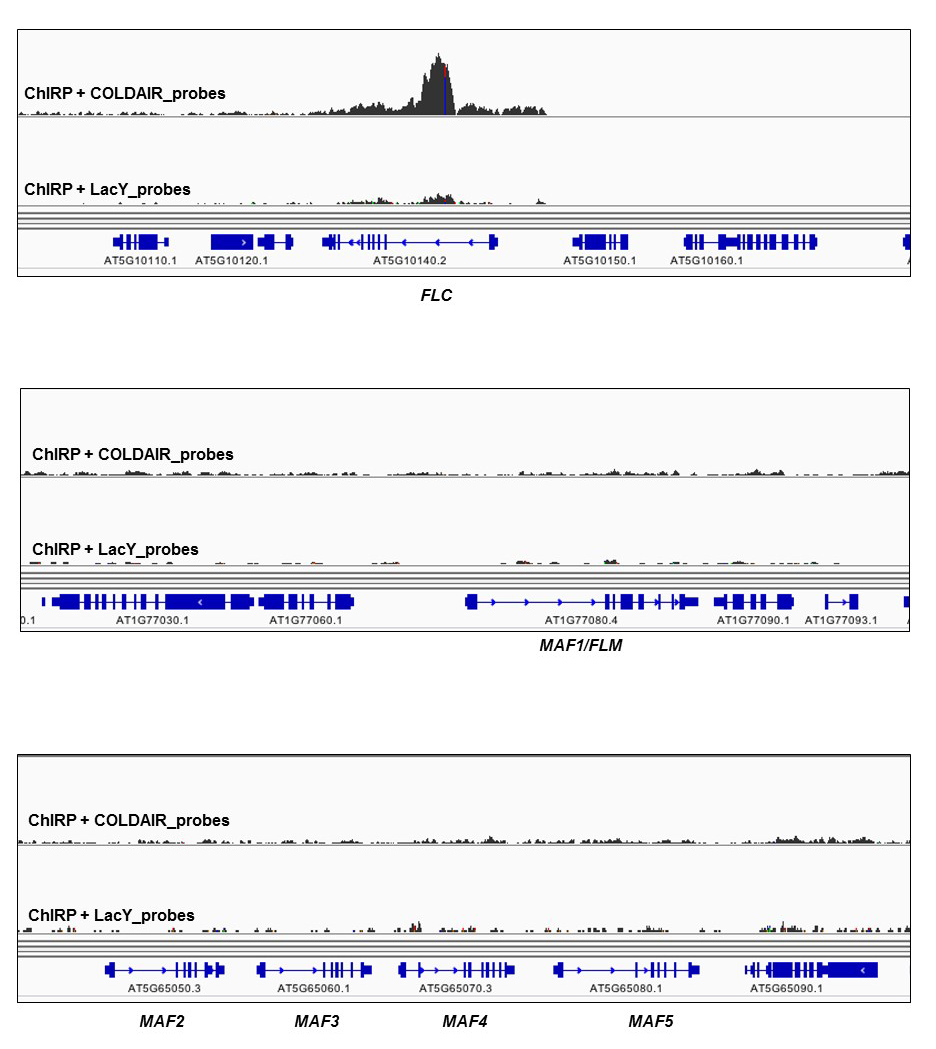

Supplement: S1 Fig — ChIRP-Seq read distribution around FLC and MAF family members (MAF1-MAF5). (JPG) [file pgen.1006939.s003.jpg]

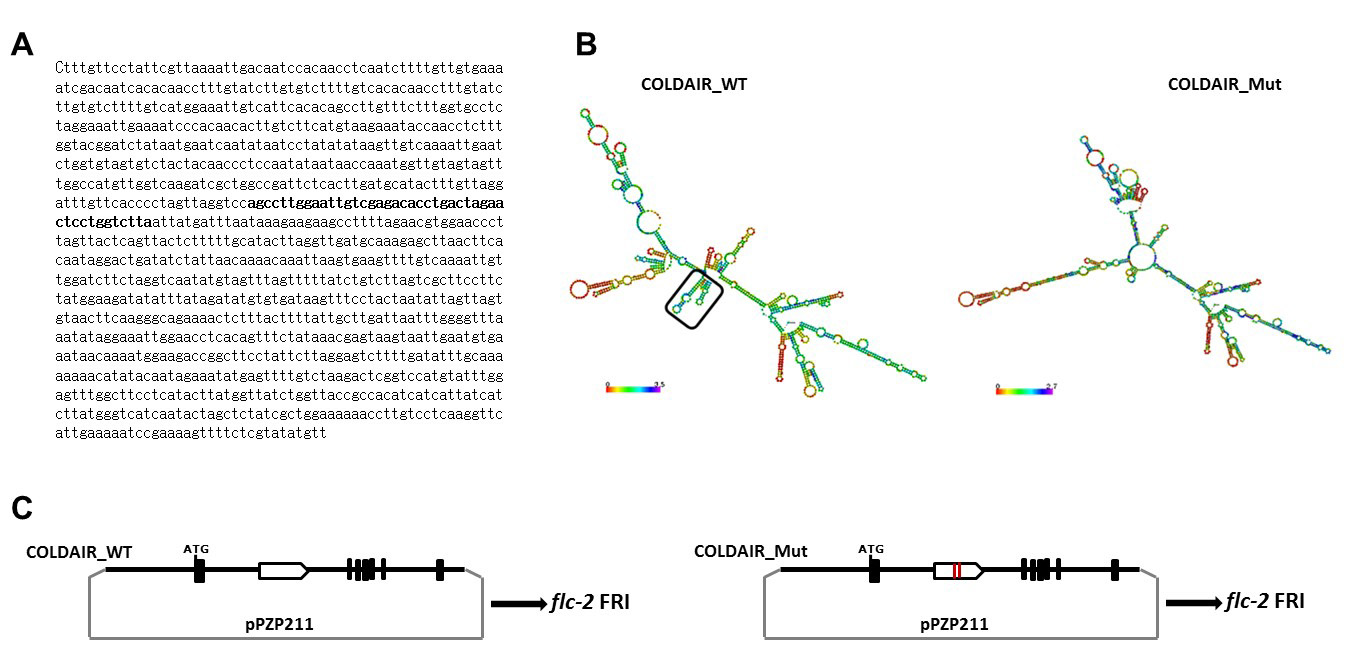

Supplement: S2 Fig — (A) The sequence of COLDAIR. Sequences that are predicted to form a stem-and-loop structure within 401–600 nucleotide region of COLDAIR are indicated by bold characters. (B) Predicted structures of COLDAIR_WT and COLDAIR_Mut. Boxed area in COLDAIR_WT shows the region shown in Fig 3A. The structural models were predicted using the RNA_fold algorithm (http://rna.tbi.univie.ac.at/cgi-bin/RNAWebSuite/RNAfold.cgi). (C) Schematic of constructs used to create COLDAIR_WT (left) and COLDAIR_Mut plants (right). These constructs are introduced into flc-2FRI_Col mutants. (JPG) [file pgen.1006939.s004.jpg]

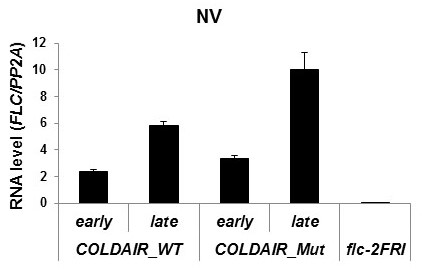

Supplement: S3 Fig — The level of FLC expression of 10 randomly selected T2-pools of transgenic lines carrying the mutant COLDAIR (COLDAIR_Mut) in flc-2 mutant background and the wild-type COLDAIR (COLDAIR_WT) at the second generation (T2) compared to the non-transgenic (flc-2FRI). Due to the FLC transgene variability, transgenic lines were grouped together based on their flowering time before vernalization. (JPG) [file pgen.1006939.s005.jpg]

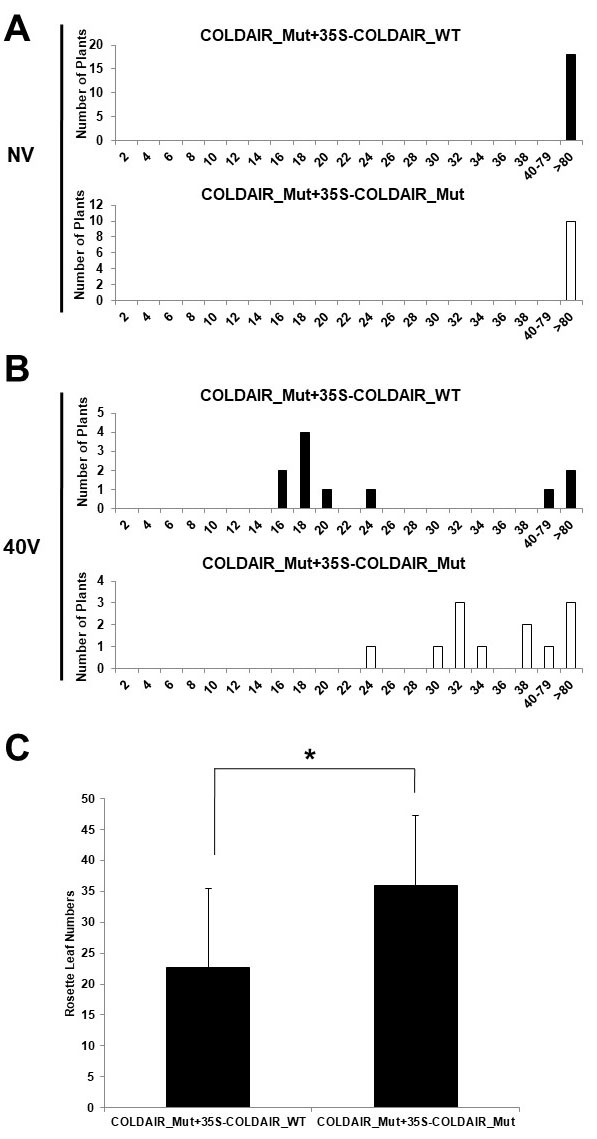

Supplement: S4 Fig — Number of COLDAIR_Mut plants complemented with the wild-type and mutant 35S::COLDAIR that flowered in NV (A) and 40V (B) conditions. X-axis: rosette leaf number at flowering. (C) flowering time of Primary transgenic plants of COLDAIR_Mut+35S-COLDAIR_WT (n = 9) and COLDAIR_Mut+35S-COLDAIR_Mut (n = 9) in 40V condition. Plants showing >80 leaves were excluded from statistical analysis because it is considered to be non-flowering. Data plotted are means ± SD; * p<0.5. (JPG) [file pgen.1006939.s006.jpg]
